# GSK-3β Contributes to Parkinsonian Dopaminergic Neuron Death: Evidence From Conditional Knockout Mice and Tideglusib

**DOI:** 10.3389/fnmol.2020.00081

**Published:** 2020-06-03

**Authors:** Junyu Li, Shanshan Ma, Jingnan Chen, Kunhua Hu, Yongyi Li, Zeyu Zhang, Zixiang Su, James R. Woodgett, Mingtao Li, Qiaoying Huang

**Affiliations:** ^1^Guangdong Provincial Key Laboratory of Brain Function and Disease, Zhongshan School of Medicine, Sun Yat-sen University, Guangzhou, China; ^2^Department of Pharmacology, Zhongshan School of Medicine, Sun Yat-sen University, Guangzhou, China; ^3^Zhixin High School, Guangzhou, China; ^4^Lunenfeld-Tanenbaum Research Institute, Sinai Health System, Toronto, ON, Canada

**Keywords:** Parkinson’s disease, GSK-3β, GSK-3α, tideglusib, neuroprotection, MPTP

## Abstract

Glycogen synthase kinase-3 (GSK-3) dysregulation has been implicated in nigral dopaminergic neurodegeneration, one of the main pathological features of Parkinson’s disease (PD). The two isoforms, GSK-3α and GSK-3β, have both been suggested to play a detrimental role in neuronal death. To date, several studies have focused on the role of GSK-3β on PD pathogenesis, while the role of GSK-3α has been largely overlooked. Here, we report *in situ* observations that both GSK-3α and GSK-3β are dephosphorylated at a negatively acting regulatory serine, indicating kinase activation, selectively in nigral dopaminergic neurons following exposure of mice to 1-methyl-4-pheny-1,2,3,6-tetrahydropyridine (MPTP). To identify whether GSK-3α and GSK-3β display functional redundancy in regulating parkinsonian dopaminergic cell death, we analysed dopaminergic neuron-specific *Gsk3a* null (*Gsk3a*^Δ*Dat*^) and *Gsk3b* null (*Gsk3b*^Δ*Dat*^) mice, respectively. We found that* Gsk3b*^Δ*Dat*^, but not *Gsk3a*^Δ*Dat*^, showed significant resistance to MPTP insult, revealing non-redundancy of GSK-3α and GSK-3β in PD pathogenesis. In addition, we tested the neuroprotective effect of tideglusib, the most clinically advanced inhibitor of GSK-3, in the MPTP model of PD. Administration of higher doses (200 mg/kg and 500 mg/kg) of tideglusib exhibited significant neuroprotection, whereas 50 mg/kg tideglusib failed to prevent dopaminergic neurodegeneration from MPTP toxicity. Administration of 200 mg/kg tideglusib improved motor symptoms of MPTP-treated mice. Together, these data demonstrate GSK-3β and not GSK-3α is critical for parkinsonian neurodegeneration. Our data support the view that GSK-3β acts as a potential therapeutic target in PD and tideglusib would be a candidate drug for PD neuroprotective therapy.

## Introduction

Parkinson’s disease (PD) is the most common neurodegenerative movement disorder, whose major clinical symptoms include resting tremor, rigidity, and bradykinesia (Emamzadeh and Surguchov, 2018). Striatal dopamine deficiency caused by the loss of nigral dopaminergic neurons is the main pathological feature of PD and responsible for the motor symptoms (Kalia and Lang, 2015). Dopamine replacement has been the first-line therapy for 40 years, but it only alleviates motor symptoms and fails to hamper dopaminergic neurodegeneration (Antonini et al., 2018). It is imperative to establish new drug targets and to develop safe and effective disease-modifying agent for treating PD.

Increasing evidence suggests that elevated activity of glycogen synthesis kinase-3β (GSK-3β) contributes to the pathogenesis of PD (Golpich et al., 2015). Expression of GSK-3β was found to be increased in the nigral pigmented neurons in postmortem PD brains (Nagao and Hayashi, 2009). GSK-3β was activated by phosphorylation at its Tyr216 in the striatum of PD patients (Duka et al., 2009; Wills et al., 2010). Increased GSK-3β protein levels have also been reported in peripheral blood lymphocytes in PD patients (Armentero et al., 2011). Our previous study showed, for the first time, that pharmacological inhibition of GSK-3β exerted neuroprotective effects in a parkinsonian model (Wang et al., 2007). Another study using different PD animal models induced by either 6-hydroxydopamine (6-OHDA) or lipopolysaccharide (LPS) also demonstrated that GSK-3β inhibition prevented dopaminergic neurodegeneration (Morales-Garcia et al., 2013). The detrimental effect of GSK-3β activation on dopaminergic neuron survival was further demonstrated in transgenic mice expressing a constitutively active mutant of GSK-3β (Credle et al., 2015). To date, cell-type-specific roles of GSK-3β in nigral dopaminergic neurodegeneration have not been determined.

GSK-3 is a pleiotropic kinase consisting of two genetically distinct isoforms, α and β, which share high similarity within the kinase domain but differ in their N- and C-terminal regions (Woodgett, 1990). GSK-3 isoforms exhibit high basal levels of activity and require stimuli for inhibition (Woodgett, 1990). Both isoforms are negatively regulated by N-terminal phosphorylation of serine residues: Ser21 in GSK-3α and Ser9 in GSK-3β (Sutherland et al., 1993; Sutherland and Cohen, 1994). Emerging evidence has shown a role for GSK-3α in brain functioning and neurodegenerative disease (Kaidanovich-Beilin et al., 2009; Hurtado et al., 2012; Morgan-Smith et al., 2014; Cymerman et al., 2015). We and other groups have shown that both GSK-3α and GSK-3β play a crucial role in neuronal death (Liang and Chuang, 2007; Leng et al., 2008; Song et al., 2010; Russell et al., 2011). Although conclusions made by previous studies focused on GSK-3β, pharmacological inhibition of GSK-3β could not distinguish the roles of the two GSK-3 isoforms.

Inhibition of GSK-3β has been considered as a potential potent option in parkinsonian therapy (Duda et al., 2018). Tideglusib, an ATP non-competitive GSK-3β inhibitor from the tiadiazolidinone family, has been tested clinically in several neurological disorders (Mathuram et al., 2018). Tideglusib treatment was associated with alleviated brain atrophy in a phase II trial of progressive supranuclear palsy (PSP), which is sometimes mistaken for PD (Höglinger et al., 2014). Tideglusib treatment also decreased levels of beta-secretase 1 (BACE-1) and phospho-tau in cerebral spinal fluid of Alzheimer’s disease patients (Lovestone et al., 2015). Moreover, tideglusib exhibited inspired benefits in a phase II trial of autism spectrum disorder (Anagnostou et al., 2018) and myotonic dystrophy (Horrigan et al., 2018). These findings imply that patients could derive beneficial effects from using tideglusib for treatment of PD. Recently, Armagen et al. proposed that tideglusib could enhance cell viability via modulation of the nuclear factor erythroid 2-related factor 2/antioxidant response element (Nrf2/ARE) pathway in an MPP^+^-induced cell damage model, implying that tideglusib might act by antagonizing parkinsonian agents (Armagan et al., 2019). However, whether tideglusib shows benefit for neuroprotection in animal models of PD has yet to be investigated.

In this study, we investigated whether both GSK-3α and GSK-3β were activated during dopaminergic neuron loss. We then used conditional knockout mice to evaluate the contributions of each isoform of GSK-3 to parkinsonian dopaminergic neuron death *in vivo*. Furthermore, we tested the neuroprotective effect of tideglusib in a mouse model of PD, in order to provide preclinical evidence for a possible disease-modifying therapy for PD.

## Materials and Methods

### Animals

All animal experiments were performed in accordance with the guidelines of the Institutional Animal Care and Use Committee of Sun Yat-sen University. The protocol was reviewed and approved by the Ethics Committee of ZSSOM on Laboratory Animal Care. The mice were housed in rooms with controlled 12 h light/dark cycles, temperature, and humidity, and food and water were provided *ad libitum*. Eight- to 12-week-old male C57BL/6 mice weighing 22–28 g were obtained from the Beijing Vital River Laboratory Animal Technological Company (Beijing, China). The dopamine transporter-driven Cre mutant (Dat-Cre) mice were obtained from The Jackson Laboratory (Stock number #006660 RRID:IMSR JAX:006660). The floxed *Gsk3a* and *Gsk3b* mice have been previously reported (MacAulay et al., 2007; Patel et al., 2008). All genetically engineered mice listed above were backcrossed to C57BL/6 for at least 10 generations.

### MPTP Treatments

The experimental procedure of MPTP treatments was performed as previously described (Huang et al., 2016; Yu et al., 2018). Briefly, 8–12-week-old male mice weighting 22–28 g were injected intraperitoneally (i.p.) with 30 mg/kg of free base MPTP-HCl (Sigma, China) at 24 h intervals for five consecutive days. The control mice received a corresponding volume of saline alone. For investigation of activation of GSK-3α and GSK-3β, mice were sacrificed 6 h after 1, 3 or 5 doses of MPTP injection (referred to as M1×, M3× or M5×, respectively). For investigation of dopaminergic lesions, mice were sacrificed 21 days post the last MPTP treatment.

For immunofluorescent or immunohistochemical analysis, mice were deeply anesthetized with choral hydrate (400 mg/kg, i.p.) and then perfused intracardially with ice-cold phosphate buffered saline (PBS), followed by 4% paraformaldehyde (PFA, Sigma, China) in PBS at a flow rate of 10 ml/min. The mice were then decapitated, brains removed and post-fixed in 4% PFA at 4°C overnight, followed by immersion in 20% and 30% sucrose.

### Tideglusib Administration

The protocol of tideglusib administration was determined according to works from Serenó et al. (2009) with modifications. Briefly, tideglusib (Selleck, China) was suspended in vehicle (26% PEG400 + 15% Chremophor EL + MilliQ) at a gradient dosage of 50 mg/kg, 200 mg/kg or 500 mg/kg. Tideglusib was orally administered for three consecutive days before MPTP injections, while control mice received a corresponding volume (10 ml/kg) of vehicle alone. During MPTP insult, gavage of tideglusib or vehicle was performed 3 h before MPTP or saline administration. Following the last MPTP or saline injection, tideglusib or vehicle was administered for another seven consecutive days. Mice were sacrificed 14 days post the last tideglusib treatment.

### Immunofluorescent Analysis

Immunofluorescent analysis was performed as previously described (Huang et al., 2016; Yu et al., 2018; Hu et al., 2019). In brief, cryostat-coronal sections (20 μm) encompassing the entire midbrain were serially collected. Free-floating sections were pre-incubated in blocking solution containing 5% normal donkey serum and 0.3% Triton X-100 in 50 mM Tris-buffered saline (TBS, pH = 7.4) at room temperature for 1 h. Primary antibodies against p-GSK-3α Ser21 (Cell Signaling Technology, Cat. #9316, RRID:AB_659836), p-GSK-3β Ser9 (Cell Signaling Technology, Cat. #5558, RRID:AB_10013750), GSK-3α (Abcam, Cat. #ab40870, RRID:AB_732666), GSK-3β (Santa Cruz, Cat. #sc-9166, RRID:AB_647604), and TH (Merck Millipore, Cat. #AB9702, RRID:AB_570923) were dissolved in diluent and incubated with sections overnight at 4°C. After washing three times, sections were incubated with the secondary antibodies (Thermo Fisher or Jackson ImmunoResearch), which were conjugated with Alexa 488 or Alexa 555 at room temperature for 1 h. Finally, the sections were visualized under a confocal laser scanning microscope (LSM 780, Carl Zeiss, Germany).

### Behavioral Tests

Behavioral tests were carried out on the 4th day following the final injection of MPTP. Two tests were used to analyze sensorimotor function of the mice.

#### Challenging Beam Test

Challenging beam test was set up as described in previous studies with minor modification (Fleming et al., 2013; Mann and Chesselet, 2015; Bhurtel et al., 2019). Briefly, a plexiglass beam consists of four equal length sections (25 cm per section, 100 cm total length) that each have a descending width (starting with 3.5 cm width and narrow to 0.5 cm at 1-cm interval). Mesh grids (1 cm^2^ squares) with width corresponding to the size of the beam were placed over the beam surface leaving a 1.5 cm space between the grid and the beam surface. Mice were trained for 4 days (five times per day) before drug administration without mesh grids. On the day of testing, the mesh grids were placed over the beam surface and mice were videotaped while traversing on the grid-surfaced beam from the widest to the narrowest section and reached their home cage for a total of five trials. Time to traverse was measured and noted in the test. Videoclips were later reviewed in slow-motion to count the number of error steps and total steps. An error step was counted when a limb slipped beyond 0.5 cm below the grid surface during a forward movement.

#### Cylinder Test

Cylinder test was carried out after the challenging beam test on the same day as described in previous studies (Fleming et al., 2013; Mann and Chesselet, 2015; Bhurtel et al., 2019). In brief, a transparent glass cylinder with a height of 20 cm and a diameter of 13 cm was used in this test. On the day of test, each mouse was placed in the cylinder for 3 min and the process was videotaped for analysis. Times for rearing were counted when the mouse raised both its forelimbs above shoulder level and put down the limbs from cylinder before another rearing.

### Immunohistochemistry

Cryo-coronal sections (40 μm) containing the midbrain and striatum were serially collected. The sections were processed for TH (Merck Millipore, Cat. #AB152, RRID:AB_390204) staining and visualized using the Vectastian Elite ABC Kit (Vector Labs, Cat. PK-6101) and the DAB peroxidase substrate kit (Vector Labs, Cat. SK-4100) following the manufacturer’s protocol. Adjacent substantia nigra sections were used for Nissl-staining to evaluate the survival of nigral neurons.

### Stereological Cell Counting and Densitometric Analysis

Stereological cell counting and densitometric analysis were performed as previously described (Huang et al., 2016; Yu et al., 2018; Hu et al., 2019). In brief, stereological cell counting analyses were performed by using the stereological method of optical fractionator with the aid of Stereo Investigator (MicroBrightField Inc., Williston, VT, USA), The numbers of TH- and Nissl-positive cells were counted in every fourth section throughout the entire extent of the midbrain. The striatal dopaminergic fibrous density was quantified based on the mean of Integrated Optical Density (IOD) of the TH immunoreactive signal. Four sections were randomly selected from those containing the striatum at the approximate level of Bregma-0.22–1.10 mm according to the mouse brain atlas (Paxinos and Franklin, 2001), and the IOD in the dorsal striatum of each section was measured on each side using ImageJ software. All analyses were performed blind to the treatments and genotypes as described (Baquet et al., 2009).

### UPLC Analysis of Striatal MPP^+^

Measurement of MPP^+^ level was conducted as previously described with modifications (Hu et al., 2019). Briefly, mice were sacrificed 90 min after a single 30 mg/kg i.p. administration of MPTP, and the bilateral striatum were dissected and weight. Tissues were homogenized and sonicated in lysis buffer which contained 5% trichloroacetic acid (Sigma, China) with 4 μg/ml 4-phenylphridine (Sigma, China) as an internal standard, followed by centrifugation for 15 min at 15,000 *g*. Chromatographic separation of supernatant was performed on an Acquity UPLC BEH C18 column (2.1 × 50 mm, 1.7 μm, Waters). The mobile phase consisted of 50 mM potassium phosphate and 0.1% with ultrapure phosphoric acid (pH = 2.5, solvent A, 92%) and acetonitrile (solvent B, 8%). MPP^+^ and 4-phenylpyridine signals were detected under PDA ultraviolet detection by Acquity Ultra Performance Liquid Chromatography H-Class system (Waters, Milford, MA, USA).

### Statistical Analysis

Statistical tests were performed using GraphPad Prism version 8.0 for Mac (GraphPad Software, La Jolla, CA, USA). All data are presented as mean ± standard error of the mean (SEM). Unpaired two-tailed *t*-tests were used for comparisons of MPP^+^ striatal level between *Gsk3a*^Δ*Dat*^ mice and their control littermates, as well as *Gsk3b*^Δ*Dat*^ mice and their control littermates. When there were two variables (genotype and treatment), one-way ANOVA or two-way ANOVA followed by Tukey’s or Holm-Sidak’s multiple comparisons test was used.

## Results

### Both GSK-3α and GSK-3β Are Activated During Parkinsonian Dopaminergic Neurodegeneration

**Figure 1:**
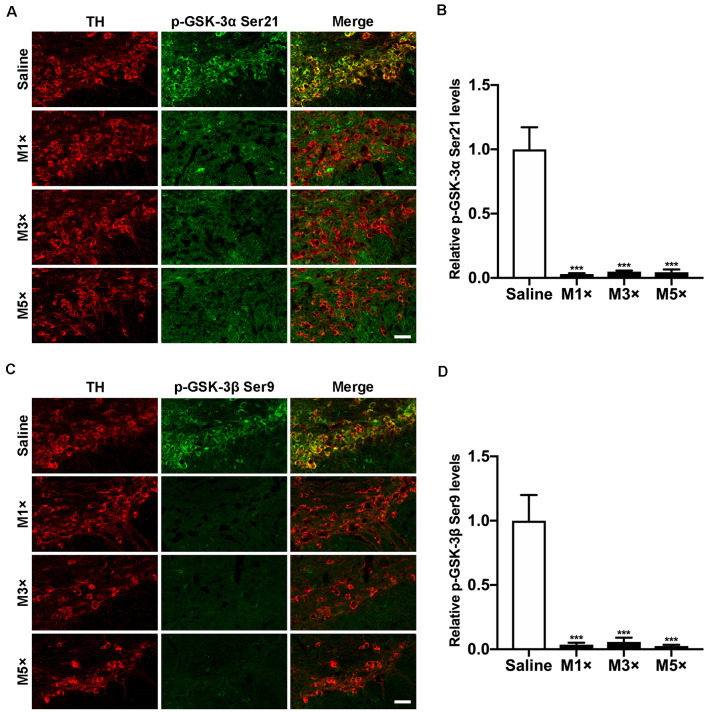
Both Glycogen synthase kinase-3α (GSK-3α) and GSK-3β are activated selectively in 1-methyl-4-pheny-1,2,3,6-tetrahydropyridine (MPTP)-treated mice. Mice were treated with 1, 3, and 5 doses of MPTP and sacrificed 6 h post-injection (M1×, M3×, M5×). **(A,B)** Immunofluorescent detection of p-GSK-3α (Ser21; green) in TH-positive dopaminergic neurons (red) by immunofluorescence **(A)** and quantitative data are shown **(B)**. Scale bar: 50 μm. Data are expressed as mean ± SEM (*n* = 4–5 per group). ***p < 0.001 vs. saline-treated mice. **(C,D)** The detection of p-GSK-3β (Ser9; green) in TH-positive dopaminergic neurons (red) by immunofluorescence **(C)** and quantitative data are shown **(D)**. Scale bar: 50 μm. Data are expressed as mean ± SEM (*n* = 4–5 per group). ***p < 0.001 vs. saline-treated mice.

Dephosphorylation of GSK-3β at Ser9, a marker indicating activation of GSK-3β, was widely observed in various PD models *in vitro* and *in vivo* (Nagao and Hayashi, 2009; Wang et al., 2013; Lin et al., 2016; Chen et al., 2017). In our previous work, we showed that activation of GSK-3β occurred selectively in dopaminergic neurons during MPTP lesion (Wang et al., 2007). However, whether GSK-3α was also activated in nigral neurons was unknown. To investigate the alteration of GSK-3α activity in parkinsonian neurodegeneration, we performed an *in situ* immunofluorescent assay for GSK-3α Ser21 phosphorylation with a specific antibody in the time course of an MPTP sub-acute model. As shown in Figure 1A, a prominent staining of GSK-3α located mainly in the dopaminergic neurons within the ventral midbrain was observed in the saline-treated group. The level of phospho-GSK-3α decreased significantly as early as 6 h after the first dose of MPTP injection, which remained constant during consecutive MPTP treatments (Figures 1A,B). We also performed a similar time course experiment for detection of phosphorylation of GSK-3β at Ser9, which was temporally and spatially consistent with that of phospho-GSK-3α (Figures 1C,D). Our immunostaining results show that and phospho-GSK-3α Ser21 and phospho-GSK-3β Ser9 are weakly detected in surrounding glial cells, while they are apparent in the nigral dopaminergic neurons, which are similar to those shown in other studies (Roh et al., 2005; Endo et al., 2006; Li et al., 2007, 2016; Gómez-Sintes and Lucas, 2010; Hurtado et al., 2012; Papazoglou et al., 2015; Wang et al., 2018; Kim et al., 2019), implying the abundance of phospho-GSK-3α Ser21 and phospho-GSK-3β Ser9 are much higher in neurons than those in glial cells. These data demonstrate that both GSK-3α and GSK-3β are activated synchronously during parkinsonian dopaminergic neurodegeneration.

### Conditional Knockout of *Gsk3a* Does Not Exert Overt Neuroprotection Against MPTP Toxicity in Mice

**Figure 2:**
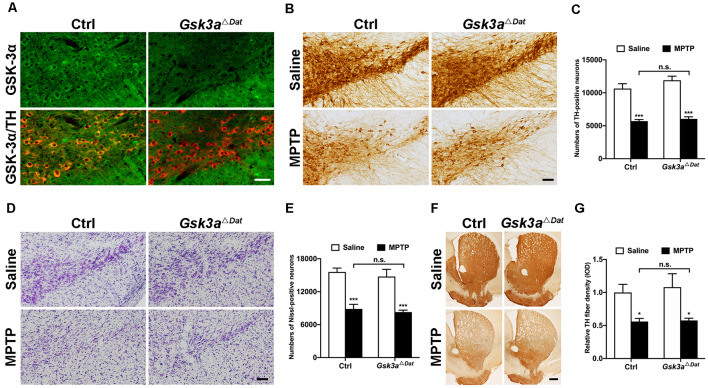
Evaluation of the role of GSK-3α in dopaminergic neurons in contribution to MPTP-induced parkinsonian neurodegeneration. **(A)** Immunofluorescence analysis for GSK-3α (green) in the ventral midbrains from MPTP-injected littermate control and *Gsk3a*^Δ*Dat*^ mice. Tyrosine hydroxylase (TH; red) staining marks dopaminergic neurons in the substantia nigra. Scale bar: 50 μm. *n* = 3 per group. **(B,C)** Immunohistochemical staining of TH on the midbrain sections from saline- and MPTP-injected control littermates and *Gsk3a*^Δ*Dat*^ mice **(A)** and cell counts of TH-positive neurons of the whole SNpc are shown **(C)**. Scale bar: 100 μm. Data show the mean ± SEM (*n* = 5–8 per group). ***p < 0.001 vs. saline-treated mice of the same genotype. n.s. no significance vs. control littermates as indicated. **(D,E)** Nissl staining on the midbrain sections from saline- and MPTP-injected control littermates and *Gsk3a*^Δ*Dat*^ mice **(D)** and cell counts of Nissl-positive neurons of the whole SNpc are shown **(E)**. Scale bar: 100 μm. Data show the mean ± SEM (*n* = 5–8 per group). ***p < 0.001 vs. saline-treated mice of the same genotype. n.s. = no significance vs. control littermates as indicated. **(F,G)** Immunohistochemical staining of TH on striatal sections from saline- and MPTP-injected control littermates and *Gsk3a*^Δ*Dat*^ mice **(F)** and measured Integrated Optical Density (IOD) of striatal TH immunostaining **(G)**. Scale bar: 500 μm. Data show the mean ± SEM (*n* = 5–8 per group). *p < 0.05 vs. saline-treated mice of the same genotype. n.s. = no significance vs. control littermates as indicated.

To evaluate whether GSK-3α contributes to dopaminergic neurodegeneration in parkinsonian lesions, we bred dopaminergic neuron-specific *Gsk3a* (*Gsk3a*^Δ*Dat*^) knockout mice by crossing Dat-Cre mice with mice expressing a floxed allele of *Gsk3a* (MacAulay et al., 2007). Cytoplasmic localization of GSK-3α in dopaminergic neurons was abolished, where tyrosine hydroxylase (TH) was used as a cell type marker, shown by immunofluorescent staining (Figure 2A). After verification of the successful conditional deletion of *Gsk3a*, we challenged the *Gsk3a*^Δ*Dat*^ mice and their control littermates with MPTP to investigate whether the nigral dopaminergic neurons of *Gsk3a*^Δ*Dat*^ mice would be resistant to MPTP toxicity. Surprisingly, *Gsk3a*^Δ*Dat*^did not show overt protection against MPTP by comparing TH-positive cell loss with control littermates (46.4% and 49.2% reductions in the control and *Gsk3a*^Δ*Dat*^ mice, respectively; Figures 2B,C). Nissl-positive cell counts showed a similar trend (43.1% and 43.9% reductions in the control and *Gsk3a*^Δ*Dat*^ mice, respectively; Figures 2D,E), revealing an actual loss of TH-positive neurons rather than reduction of TH expression. Depletion of striatal dopaminergic fiber density (44.0% and 50.5% reductions of TH-immunoreactive signal in the control and *Gsk3a*^Δ*Dat*^ mice, respectively; Figures 2F,G) revealed similar trends and statistical results in reduction of nigral neurons. The striatal MPP^+^ level assay showed that there was no difference between *Gsk3a*^Δ*Dat*^ mice and their control littermates, indicating MPTP metabolism was not altered between these mice (Table 1). These data demonstrate that selective elimination of GSK-3α in dopaminergic neurons exhibits no significant nigro-striatal neuroprotection against MPTP toxicity.

**Table 1:** Conditional knockout of *Gsk3a* in dopaminergic neurons (*Gsk3a*^Δ*Dat*^) does not alter brain metabolism of 1-methyl-4-pheny-1,2,3,6-tetrahydropyridine (MPTP).

Mice	MPP^+^ (μg/g)
Control littermates	9.50 ± 2.35
*Gsk3a*^Δ*Dat*^	12.2 ± 0.64

*Values of MPP^+^, in units of μg/g, represent mean ± SEM for 3–4 mice per group. *p* = 0.7876 for *Gsk3a*^Δ*Dat*^ vs. control littermates*.

### Conditional Knockout of *Gsk3b* Rescues Dopaminergic Neurodegeneration in an MPTP Mouse Model of PD

**Figure 3:**
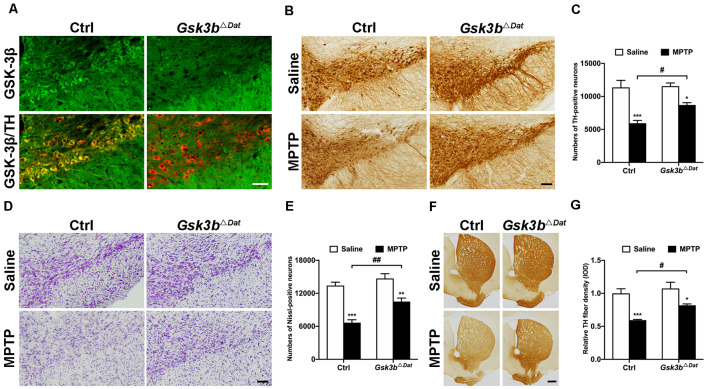
Dopaminergic neuron-associated GSK-3β contributes to MPTP-induced parkinsonian neurodegeneration. **(A)** Immunofluorescence analysis for GSK-3β (green) in the ventral midbrains from MPTP-injected littermate control and *Gsk3b*^Δ*Dat*^ mice. TH (red) staining marks dopaminergic neurons in the substantia nigra. Scale bar: 50 μm. *n* = 3 per group. **(B,C)** Immunohistochemical staining of TH on the midbrain sections from saline- and MPTP-injected control littermates and *Gsk3b*^Δ*Dat*^ mice **(A)** and cell counts of TH-positive neurons of the whole SNpc are shown **(C)**. Scale bar: 100 μm. Data show the mean ± SEM (*n* = 5–6 per group). *p < 0.05, ***p < 0.001 vs. saline-treated mice of the same genotype. ^#^*p* < 0.05 vs. control littermates as indicated. **(D,E)** Nissl staining on the midbrain sections from saline- and MPTP-injected control littermates and *Gsk3b*^Δ*Dat*^ mice **(D)** and cell counts of Nissl-positive neurons of the whole SNpc are shown **(E)**. Scale bar: 100 μm. Data show the mean ± SEM (*n* = 5–6 per group). **p < 0.01, ***p < 0.001 vs. saline-treated mice of the same genotype. ^##^*p* < 0.01 vs. control littermates as indicated. **(F,G)** Immunohistochemical staining of TH on striatal sections from saline- and MPTP-injected control littermates and *Gsk3b*^Δ*Dat*^ mice **(F)** and measured IOD of striatal TH immunostaining **(G)**. Scale bar: 500 μm. Data show the mean ± SEM (*n* = 5–6 per group). *p < 0.05, ***p < 0.001 vs. saline-treated mice of the same genotype. ^#^*p* < 0.05 vs. control littermates as indicated.

To clarify the precise role of GSK-3β in dopaminergic neurodegeneration, we generated dopaminergic neuron-specific *Gsk3b* knockout mice (*Gsk3b*^Δ*Dat*^) by crossing Dat-Cre mice with mice expressing a floxed allele of *Gsk3b* (Patel et al., 2008). Successful conditional deletion of GSK-3β expression in dopaminergic neurons was confirmed by immunofluorescent staining (Figure 3A). MPTP was administered to the *Gsk3b*^Δ*Dat*^ mice and their control littermates to determine whether the nigral dopaminergic neurons of *Gsk3b*^Δ*Dat*^ mice would be refractory to MPTP insult. In agreement with our expectation, *Gsk3b*^Δ*Dat*^ mice showed a lower reduction of both the nigral TH-positive cell bodies (47.7% and 24.7% reductions in the control and *Gsk3b*^Δ*Dat*^ mice, respectively; Figures 3B,C) and Nissl staining cell counts (50.3% and 28.6% reductions in the control and *Gsk3b*^Δ*Dat*^ mice, respectively; Figures 3D,E) compared with the control littermates. The decrease of striatal TH-positive fibers density was consistent with the reduction in the numbers of SNpc neurons (40.6% and 25.5% reductions in the control and *Gsk3b*^Δ*Dat*^ mice, respectively; Figures 3F,G). These data demonstrated that GSK-3β in dopaminergic neurons does play an important role in parkinsonian neurodegeneration. We also performed analysis of striatal MPP^+^ levels between *Gsk3b*^Δ*Dat*^ mice and their control littermates, the result of which showed that MPTP metabolism was not altered between these mice (Table 2). These results demonstrate that selective elimination of GSK-3β in dopaminergic neurons exhibits significant nigro-striatal neuroprotection against MPTP toxicity.

**Table 2:** Conditional knockout of *Gsk3b* in dopaminergic neurons (*Gsk3b*^Δ*Dat*^) does not alter brain metabolism of MPTP.

Mice	MPP^+^ (μg/g)
Control littermates	13.53 ± 1.29
*Gsk3b*^Δ*Dat*^	12.33 ± 1.47

*Values of MPP^+^, in units of μg/g, represent mean ± SEM for 3–4 mice per group. *p* = 0.5627 for *Gsk3b*^Δ*Dat*^ vs. control littermates*.

### GSK-3 Inhibitor Tideglusib Alleviates Loss of Nigral Dopaminergic Neurons in the Parkinsonian Mice

**Figure 4:**
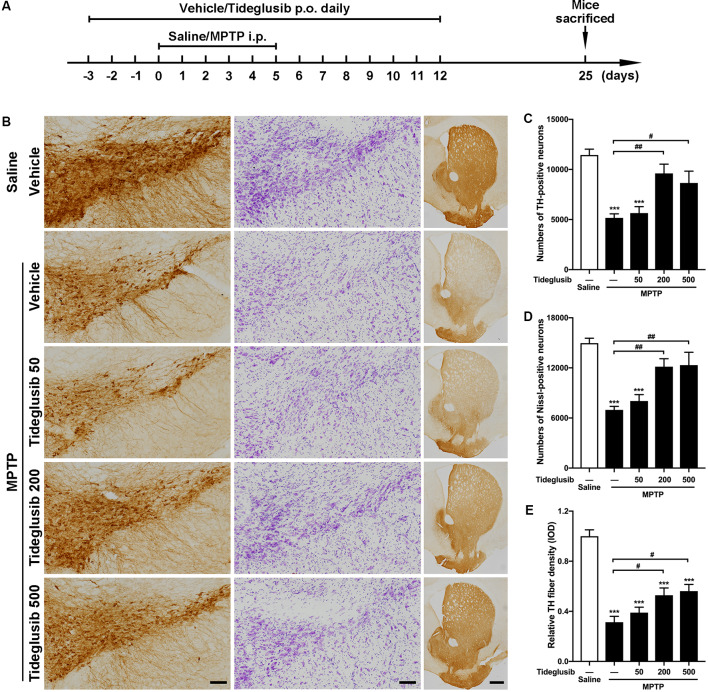
Tideglusib administration exerts neuroprotection against MPTP lesions. **(A)** A graphic of the experimental procedure is shown. **(B)** Immunohistochemical staining of TH (left panel), Nissl (middle panel) on the midbrain sections and TH staining on striatal sections (right panel) from saline- and MPTP-injected control and mice with gradient dosages of Tideglusib (50 mg/kg, 200 mg/kg, and 500 mg/kg). Scale bar: 100 μm (Midbrain), 500 μm (Striatum). *n* = 6–8 per group. **(C–E)** The bar graph shows the cell counts of TH-positive neurons of the whole SNpc **(C)**, Nissl-positive cells of the whole SNpc **(D)**, and measured IOD of striatal TH immunostaining **(E)**. The data shows the mean ± SEM (*n* = 6–8 per group). ***p < 0.001 vs. saline-treated mice with vehicle administration. ^#^*p* < 0.05, ^##^*p* < 0.01 vs. MPTP mice with vehicle administration as indicated.

We next tested the therapeutic potential of the GSK3β inhibitor, tideglusib, to rescue parkinsonian neurodegeneration provoked by MPTP toxicity. A gradient of three doses of tideglusib were given by oral gavage. A graphic of the experimental procedure is shown in Figure 4A. As expected, MPTP induced a 54.8% reduction of TH-positive neurons in the vehicle-treated groups. Administration of 200 mg/kg tideglusib significantly alleviated TH-positive neuron loss in the SNpc (16.0% reduction compared with saline mice), while 50 mg/kg tideglusib showed no therapeutic effect (50.7% reduction compared with saline mice) and 500 mg/kg tideglusib showed no better neuroprotection than 200 mg/kg (24.4% reduction compared with saline mice; Figures 4B,C). Nissl-positive cell counting confirmed the actual neuroprotection by tideglusib rather than a rescue of TH expression (53.4%, 46.3%, 18.8%, and 17.6% reductions in vehicle, 50 mg/kg, 200 mg/kg, and 500 mg/kg tideglusib treatment mice compared with saline mice, respectively; Figures 4B,D). We also observed a similar neuroprotective effect in the striatum where the TH-staining indicated the dopaminergic fiber density (68.7%, 61.0%, 47.7%, and 43.9% reduction in vehicle, 50 mg/kg, 200 mg/kg, and 500 mg/kg tideglusib treatment mice compared with saline mice, respectively; Figures 4B,E). The metabolism of MPTP was not affected by administration of tideglusib or vehicle, as shown by measurement of striatal MPP^+^ levels (Table 3). These data demonstrate the neuroprotective effects of tideglusib in the MPTP mouse model of PD.

**Table 3:** Blank refers to MPTP mice without administration of either vehicle or tideglusib; Vehicle refers to MPTP mice with vehicle administration; Tideglusib refers to MPTP mice with 200 mg/kg tideglusib administration.

Mice	MPP^+^ (μg/g)
Blank	17.02 ± 1.47
Vehicle	22.95 ± 1.97
Tideglusib	18.92 ± 2.18

*Neither vehicle nor tideglusib administration alters the brain metabolism of MPTP. Values of MPP^+^, in units of μg/g, represent mean ± SEM for 4–5 mice per group. *p* = 0.1506 for blank vs. vehicle, *p* = 0.7723 for blank vs. tideglusib, *p* = 0.3462 for vehicle vs. tideglusib, by one-way ANOVA and Tukey’s multiple comparisons test*.

### Tideglusib Improves Motor Symptoms of the MPTP-Treated Mice

**Figure 5:**
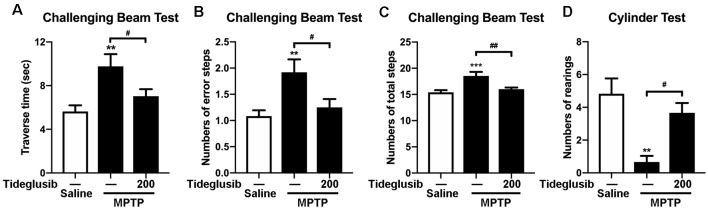
Tideglusib administration improved motor dysfunction caused by MPTP lesions. **(A–C)** The bar graphs show the results of challenging beam tests: traverse time **(A)**, Numbers of error steps **(B)**, and numbers of total steps **(C)**, respectively. The data shows the mean ± SEM (*n* = 10–12 per group). **p < 0.01, ***p < 0.001 vs. saline-treated mice with vehicle administration. ^#^*p* < 0.05, ^##^*p* < 0.01 vs. MPTP mice with vehicle administration as indicated. **(D)** The bar graph shows the numbers of rearings in the cylinder test. The data shows the mean ± SEM (*n* = 10–12 per group). **p < 0.01 vs. saline-treated mice with vehicle administration. ^#^*p* < 0.05 vs. MPTP mice with vehicle administration as indicated.

To ensure the neuroprotective effect of tideglusib in MPTP mouse model, we investigated whether tideglusib (200 mg/kg) could ameliorate motor deficits caused by MPTP treatment. Challenging beam test was used to assess changes in basal ganglia function of the mice. In vehicle groups, MPTP-treated mice took a longer time to reach the home cage (5.76 s and 9.76 s in saline and MPTP group respectively; Figure 5A), had increased numbers of error steps (1.08 and 1.96 error steps in saline and MPTP group respectively; Figure 5B) and had increased numbers of total steps (15.41 and 18.54 total steps in saline and MPTP group respectively; Figure 5C). Tideglusib administration significantly ameliorated these motor deficits induced by MPTP toxicity (7.03 s to reach the home cage, 1.25 error steps and 16.02 total steps; Figures 5A–C). Cylinder test was used to assess the locomotor activity of the mice. The number of rearings was reduced in MPTP-intoxicated mice compared to the saline-treated mice in vehicle groups, while MPTP with tideglusib administration increased the amount of rearing compared with MPTP with vehicle admininstration (4.83, 0.67 and 3.67 times of rearing in saline+vehicle, MPTP+vehicle and MPTP+tideglusib respectively; Figure 5D). These data demonstrate that tideglusib administration improves motor performance and coordination, as well as spontaneous movements in MPTP mouse model of PD.

## Discussion

This is the first study to analyze the impact of manipulating individual GSK-3 isoforms on nigral dopaminergic neuron survival in a mouse model of PD. We demonstrated that GSK-3β but not GSK-3α contributes to nigral dopaminergic neurodegeneration. This is also the first study using genetic ablation to demonstrate the detrimental role of GSK-3β in PD pathogenesis. Moreover, our findings indicate a beneficial effect of tideglusib on parkinsonian neuronal loss and a potential drug for disease-modifying therapy for PD.

Our results imply GSK-3α and β might be involved in the same signal pathway in MPTP-intoxicated nigral dopaminergic neurons since there is consistency of the time and degree of N-terminal serine dephosphorylation and the cellular distribution of both isoforms. It is interesting to find that *Gsk3a*^Δ*Dat*^ mice did not show overt neuroprotection against MPTP while *Gsk3b*^Δ*Dat*^ animals did show neuroprotection. This could be due to differential abundance of the expression of GSK-3α and GSK-3β, provided that the two isoforms were functionally redundant in dopaminergic neurons. We tried to ascertain the relative levels of protein expression of both isoforms by using an antibody recognizing the homologous domain of GSK-3α/β (Millipore Cat. #05-412, RRID:AB_309720). We found that the immunofluorescent signal from this antibody within the dopaminergic neurons almost disappeared in *Gsk3b*^Δ*Dat*^ mice but mostly stayed the same in *Gsk3a*^Δ*Dat*^ mice, suggesting GSK-3β was the major isoform expressed in dopaminergic neurons (data not shown). Although, we have verified that the antibody has the same affinity for either isoform in western blot by using tagged proteins, it has not been confirmed that the affinity is the same in the immunofluorescent assay, where the conformation of the antigens should be completely different from those in western blot. So far, there is still a lack of evidence on the abundance of GSK-3 isoforms at protein levels in dopaminergic neurons, which needs further investigation.

However, a difference in relative abundance of GSK-3α/β may not be a reasonable explanation, especially in the case that these isoforms are actually paralogs, which are homologous proteins derived from different genes. Growing evidence supports the notion that GSK-3α and GSK-3β are non-redundant (for a review see Kaidanovich-Beilin and Woodgett, 2011). Data from studies in cultured neurons indicates differential roles for GSK-3α and GSK-3β in neuronal death, as they appear to be stimuli-specific. For example, blockade of either isoform was sufficient to hamper excitotoxic neuronal death regardless of the neuronal types (Liang and Chuang, 2007; Leng et al., 2008). In rat cerebellar granule neurons, both isoforms contribute to depolarization-induced apoptosis (Song et al., 2010), while only GSK-3β promotes trophic-deprivation-induced death (Hongisto et al., 2008). Our results presented above show for the first time in a neurodegenerative model *in vivo* that GSK-3β, but not GSK-3α, promotes parkinsonian dopaminergic neuronal death. These results support non-redundancy of GSK-3 isoforms under certain conditions. The key to understanding the promiscuity of GSK-3 isoforms in multiple paradigms of neuronal death will be to further elucidate the signal pathways involved and the responsible substrates, as suggested in a recent review (Beurel et al., 2015).

As GSK-3β of dopaminergic neurons contributes to cell death in parkinsonian pathogenesis, further studies are required to clarify the underlying mechanisms. Dysregulation of GSK-3β results in aberrant mitochondrial function, which is implicated in PD (Yang et al., 2017). In our recent work, we illustrated that upregulation of Bcl-2 interacting mediator of cell death (Bim), a pro-apoptotic protein related with mitochondria-dependent cell death, in dopaminergic neurons promotes neurodegeneration in MPTP model (Hu et al., 2019). Interestingly, some evidences suggested that GSK-3β promotes Bim upregulation and subsequent cell death *in vitro* (Nuutinen et al., 2009; Rubio-Patiño et al., 2012). We wonder if GSK-3β may involve in Bim upregulation here and subsequently degenerating nigral dopaminergic neurons. Other putative mechanisms will also be considered in our future works. For example, GSK-3β may contribute to the formation of protein aggregates or intracellular inclusions in PD. Deficiency of autophagy-lysosomal pathway, leading to dysfunction of protein aggregate clearance, was observed in post-mortem brains of PD patients and PD animal models (Meredith et al., 2002; Crews et al., 2010; Dehay et al., 2010; Vila et al., 2011; Bové et al., 2014). Increasing evidence suggests that GSK-3β inhibition restored lysosomal acidification and biogenesis by mediating nuclear translocation of Transcription Factor EB (TFEB) or through GSK-3β/tuberous sclerosis complex (TSC) axis, and thus showed neuroprotective effects in pathological conditions (Avrahami et al., 2013, 2020; Azoulay-Alfaguter et al., 2015; Marchand et al., 2015; Li et al., 2016; Ren et al., 2018). In addition, some substrates, such as α-synuclein and Tau, whose phosphorylation by GSK-3β may progressively lead to intracellular and axonal deposit, may be involved in GSK-3β-mediated parkinsonian pathophysiology (Credle et al., 2015). Therefore, further investigation could focus on the role of GSK-3β in autophagy-lysosomal pathway and identification of novel substrates for aberrant protein aggregates in PD. GSK-3β also plays an important role in neuroinflammation (Beurel, 2011; Duda et al., 2018), which is responsible for neurodegeneration in PD (Gelders et al., 2018). Despite activation of GSK-3β was mainly observed in dopaminergic neurons in MPTP model, we did not rule out the possibility of GSK-3β of the surrounding glial cells mediating neuroinflammation. Substantial evidence suggests that GSK-3β mediates glial cell activation and promotes release of pro-inflammatory cytokines via regulating several transcriptional factors (e.g., NF-κB, CREB, STAT3) (Martin et al., 2005; Kim et al., 2007; Beurel and Jope, 2008; Yuskaitis and Jope, 2009; Cao et al., 2017). However, whether and how GSK-3β regulates neuroinflammation in parkinsonian pathogenesis remains unclear, which needs further investigation.

GSK-3 inhibition has been considered a potential therapeutic strategy for PD treatment (Duda et al., 2018). Our results from GSK-3 isoform-specific knockout mice suggest a GSK-3β-specific inhibitor might be a better choice for neuroprotection in PD. Although numerous GSK-3 inhibitors have been exploited, it remains challenging to develop an isoform-specific inhibitor for GSK-3β (Cormier and Woodgett, 2017). There are some data that suggest tideglusib has somewhat higher efficacy of inhibition of GSK-3β than of GSK-3α (Domínguez et al., 2012; Noori et al., 2019). Tideglusib has also shown acceptable safety and was well tolerated in several chronic clinical trials (del Ser et al., 2013; Höglinger et al., 2014; Tolosa et al., 2014; Lovestone et al., 2015; Anagnostou et al., 2018; Horrigan et al., 2018). Thus, it was introduced as the most clinically advanced inhibitor of GSK-3 (Gunosewoyo et al., 2017). Therefore, we suggest tideglusib could be a promising drug in PD treatment. As is shown in this study, tideglusib exerted significant neuroprotection and improved motor symptoms against the parkinsonian toxin MPTP. It is notable that tideglusib failed in a Phase II clinical trial of AD due to no clinical benefits in cognitive improvement (del Ser et al., 2013; Lovestone et al., 2015), despite its neuroprotection in AD animal models (Serenó et al., 2009). It has been suggested that intervention at an earlier disease stage, longer duration of treatment and better dosing of tideglusib should be taken into account for future clinical trials (Lovestone et al., 2015; Matsunaga et al., 2019). These suggestions should also be considered in clinical trials for PD, which may possibly be confronted with similar problems.

In conclusion, we have demonstrated that GSK-3β rather than GSK-3α in dopaminergic neurons is critical for parkinsonian degeneration, even with simultaneous activation of both isoforms occurring in nigral neurons during MPTP challenge. Moreover, pharmacological inhibition of GSK-3β by tideglusib alleviated parkinsonian lesions in an MPTP mouse model of PD. Our findings support the view that GSK-3β acts as a potential therapeutic target in PD, as well as provide preclinical data in support of tideglusib for PD treatment. We expect that tideglusib, or a potent, selective GSK-3β-specific inhibitor, would have potential applications in PD therapy.

## Data Availability Statement

The datasets generated for this study are available on request to the corresponding author.

## Ethics Statement

The animal study was reviewed and approved by Ethics Committee of ZSSOM on Laboratory Animal Care.

## Author Contributions

JL, QH and SM designed the experiments and performed data analysis. JL, QH and JC drafted the manuscript. JL, KH and YL performed animal models establishment, sample collection, immunofluorescent and immunohistochemistry staining. ZZ and ZS performed UPLC assay, stereological cell counting, genetic mice housing and genotyping. JL and ZS performed behavioral tests. SM revised the manuscript. JW generated *Gsk3a* and *Gsk3b* floxed mice, revised the manuscript. ML and QH supervised progression, approved the final version of manuscript and communicated with editors.

## Conflict of Interest

The authors declare that the research was conducted in the absence of any commercial or financial relationships that could be construed as a potential conflict of interest.

## References

[B1] Anagnostou E. Bennett T. A. Thorpe K. Nicolson R. (2018). 5.16 A phase 2 randomized, placebo-controlled trial of tideglusib, an orally administered GSK-3 β inhibitor, in the treatment of adolescents with ASD. J. Am. Acad. Child Adolesc. Psychiatry 57:S232 10.1016/j.jaac.2018.09.311

[B2] Antonini A. Moro E. Godeiro C. Reichmann H. (2018). Medical and surgical management of advanced Parkinson’s disease. Mov. Disord. 33, 900–908. 10.1002/mds.27340 29570862

[B3] Armagan G. Sevgili E. Gurkan F. T. Kose F. A. Bilgic T. Dagci T.. (2019). Regulation of the Nrf2 pathway by glycogen synthase kinase-3β in MPP^+^-induced cell damage. Molecules 24:E1377. 10.3390/molecules24071377 30965670 PMC6480928

[B4] Armentero M. T. Sinforiani E. Ghezzi C. Bazzini E. Levandis G. Ambrosi G.. (2011). Peripheral expression of key regulatory kinases in Alzheimer’s disease and Parkinson’s disease. Neurobiol. Aging 32, 2142–2151. 10.1016/j.neurobiolaging.2010.01.004 20106550

[B5] Avrahami L. Farfara D. Shaham-Kol M. Vassar R. Frenkel D. Eldar-Finkelman H. (2013). Inhibition of glycogen synthase kinase-3 ameliorates β-amyloid pathology and restores lysosomal acidification and mammalian target of rapamycin activity in the Alzheimer disease mouse model: *in vivo* and *in vitro* studies. J. Biol. Chem. 288, 1295–1306. 10.1074/jbc.m112.409250 23155049 PMC3543013

[B6] Avrahami L. Paz R. Dominko K. Hecimovic S. Bucci C. Eldar-Finkelman H. (2020). GSK-3-TSC axis governs lysosomal acidification through autophagy and endocytic pathways. Cell. Signal. 71:109597. 10.1016/j.cellsig.2020.109597 32173369

[B7] Azoulay-Alfaguter I. Elya R. Avrahami L. Katz A. Eldar-Finkelman H. (2015). Combined regulation of mTORC1 and lysosomal acidification by GSK-3 suppresses autophagy and contributes to cancer cell growth. Oncogene 34, 4613–4623. 10.1038/onc.2014.390 25500539

[B8] Baquet Z. C. Williams D. Brody J. Smeyne R. J. (2009). A comparison of model-based (2D) and design-based (3D) stereological methods for estimating cell number in the substantia nigra pars compacta (SNpc) of the C57BL/6J mouse. Neuroscience 161, 1082–1090. 10.1016/j.neuroscience.2009.04.031 19376196 PMC2705113

[B9] Beurel E. (2011). Regulation by glycogen synthase kinase-3 of inflammation and T cells in CNS diseases. Front. Mol. Neurosci. 4:18. 10.3389/fnmol.2011.00018 21941466 PMC3171068

[B11] Beurel E. Grieco S. F. Jope R. S. (2015). Glycogen synthase kinase-3 (GSK3): regulation, actions, and diseases. Pharmacol. Ther. 148, 114–131. 10.1016/j.pharmthera.2014.11.016 25435019 PMC4340754

[B10] Beurel E. Jope R. S. (2008). Differential regulation of STAT family members by glycogen synthase kinase-3. J. Biol. Chem. 283, 21934–21944. 10.1074/jbc.m802481200 18550525 PMC2494932

[B12] Bhurtel S. Katila N. Srivastav S. Neupane S. Choi D. Y. (2019). Mechanistic comparison between MPTP and rotenone neurotoxicity in mice. Neurotoxicology 71, 113–121. 10.1016/j.neuro.2018.12.009 30605763

[B13] Bové J. Martinez-Vicente M. Dehay B. Perier C. Recasens A. Bombrun A.. (2014). BAX channel activity mediates lysosomal disruption linked to Parkinson disease. Autophagy 10, 889–900. 10.4161/auto.28286 24686337 PMC5119069

[B14] Cao Q. Karthikeyan A. Dheen S. T. Kaur C. Ling E. A. (2017). Production of proinflammatory mediators in activated microglia is synergistically regulated by Notch-1, glycogen synthase kinase (GSK-3β) and NF-κB/p65 signalling. PLoS One 12:e0186764. 10.1371/journal.pone.0186764 29049420 PMC5648239

[B15] Chen L. Cheng L. Wei X. Yuan Z. Wu Y. Wang S.. (2017). Tetramethylpyrazine analogue CXC195 protects against dopaminergic neuronal apoptosis *via* activation of PI3K/Akt/GSK3β signaling pathway in 6-OHDA-induced Parkinson’s disease mice. Neurochem. Res. 42, 1141–1150. 10.1007/s11064-016-2148-x 28005221

[B16] Cormier K. W. Woodgett J. R. (2017). Recent advances in understanding the cellular roles of GSK-3. F1000Res 6:167. 10.12688/f1000research.10557.1 28299185 PMC5321126

[B17] Credle J. J. George J. L. Wills J. Duka V. Shah K. Lee Y. C.. (2015). GSK-3β dysregulation contributes to parkinson’s-like pathophysiology with associated region-specific phosphorylation and accumulation of tau and α-synuclein. Cell Death Differ. 22, 838–851. 10.1038/cdd.2014.179 25394490 PMC4392080

[B18] Crews L. Spencer B. Desplats P. Patrick C. Paulino A. Rockenstein E.. (2010). Selective molecular alterations in the autophagy pathway in patients with Lewy body disease and in models of α-synucleinopathy. PLoS One 5:e9313. 10.1371/journal.pone.0009313 20174468 PMC2824828

[B19] Cymerman I. A. Gozdz A. Urbanska M. Milek J. Dziembowska M. Jaworski J. (2015). Structural plasticity of dendritic spines requires GSK3α and GSK3β. PLoS One 10:e0134018. 10.1371/journal.pone.0134018 26207897 PMC4514647

[B20] Dehay B. Bove J. Rodriguez-Muela N. Perier C. Recasens A. Boya P.. (2010). Pathogenic lysosomal depletion in Parkinson’s disease. J. Neurosci. 30, 12535–12544. 10.1523/JNEUROSCI.1920-10.2010 20844148 PMC6633458

[B21] del Ser T. Steinwachs K. C. Gertz H. J. Andres M. V. Gomez-Carrillo B. Medina M.. (2013). Treatment of Alzheimer’s disease with the GSK-3 inhibitor tideglusib: a pilot study. J. Alzheimers Dis. 33, 205–215. 10.3233/jad-2012-120805 22936007

[B22] Domínguez J. M. Fuertes A. Orozco L. del Monte-Millan M. Delgado E. Medina M. (2012). Evidence for irreversible inhibition of glycogen synthase kinase-3β by tideglusib. J. Biol. Chem. 287, 893–904. 10.1074/jbc.M111.306472 22102280 PMC3256883

[B23] Duda P. Wiśniewski J. Wójtowicz T. Wójcicka O. Jaśkiewicz M. Drulis-Fajdasz D.. (2018). Targeting GSK3 signaling as a potential therapy of neurodegenerative diseases and aging. Expert Opin. Ther. Targets 22, 833–848. 10.1080/14728222.2018.1526925 30244615

[B24] Duka T. Duka V. Joyce J. N. Sidhu A. (2009). α-synuclein contributes to GSK-3β-catalyzed Tau phosphorylation in Parkinson’s disease models. FASEB J. 23, 2820–2830. 10.1096/fj.08-120410 19369384 PMC2796901

[B25] Emamzadeh F. N. Surguchov A. (2018). Parkinson’s disease: biomarkers, treatment, and risk factors. Front. Neurosci. 12:612. 10.3389/fnins.2018.00612 30214392 PMC6125353

[B26] Endo H. Nito C. Kamada H. Nishi T. Chan P. H. (2006). Activation of the Akt/GSK3β signaling pathway mediates survival of vulnerable hippocampal neurons after transient global cerebral ischemia in rats. J. Cereb. Blood Flow Metab. 26, 1479–1489. 10.1038/sj.jcbfm.9600303 16538228

[B27] Fleming S. M. Ekhator O. R. Ghisays V. (2013). Assessment of sensorimotor function in mouse models of Parkinson’s disease. J. Vis. Exp. 76:e50303. 10.3791/50303 23851663 PMC3727502

[B28] Gelders G. Baekelandt V. van der Perren A. (2018). Linking neuroinflammation and neurodegeneration in Parkinson’s disease. J. Immunol. Res. 2018:4784268. 10.1155/2018/4784268 29850629 PMC5926497

[B29] Golpich M. Amini E. Hemmati F. Ibrahim N. M. Rahmani B. Mohamed Z.. (2015). Glycogen synthase kinase-3 β (GSK-3β) signaling: implications for Parkinson’s disease. Pharmacol. Res. 97, 16–26. 10.1016/j.phrs.2015.03.010 25829335

[B30] Gómez-Sintes R. Lucas J. J. (2010). NFAT/Fas signaling mediates the neuronal apoptosis and motor side effects of GSK-3 inhibition in a mouse model of lithium therapy. J. Clin. Invest. 120, 2432–2445. 10.1172/jci37873 20530871 PMC2898581

[B31] Gunosewoyo H. Yu L. Munoz L. Kassiou M. (2017). Kinase targets in CNS drug discovery. Future Med. Chem. 9, 303–314. 10.4155/fmc-2016-0214 28176536

[B32] Höglinger G. U. Huppertz H. J. Wagenpfeil S. Andres M. V. Belloch V. León T.. (2014). Tideglusib reduces progression of brain atrophy in progressive supranuclear palsy in a randomized trial. Mov. Disord. 29, 479–487. 10.1002/mds.25815 24488721

[B33] Hongisto V. Vainio J. C. Thompson R. Courtney M. J. Coffey E. T. (2008). The Wnt pool of glycogen synthase kinase 3β is critical for trophic-deprivation-induced neuronal death. Mol. Cell. Biol. 28, 1515–1527. 10.1128/mcb.02227-06 18195042 PMC2258793

[B34] Horrigan J. McMorn A. Snape M. Nikolenko N. Gomes T. Lochmuller H. (2018). AMO-02 (tideglusib) for the treatment of congenital and childhood onset myotonic dystrophy type 1. Neuromuscular Disord. 28:S14 10.1016/s0960-8966(18)30330-4 32942085

[B35] Hu K. Huang Q. Liu C. Li Y. Liu Y. Wang H.. (2019). c-Jun/Bim upregulation in dopaminergic neurons promotes neurodegeneration in the MPTP mouse model of Parkinson’s disease. Neuroscience 399, 117–124. 10.1016/j.neuroscience.2018.12.026 30590105

[B36] Huang Q. Du X. He X. Yu Q. Hu K. Breitwieser W.. (2016). JNK-mediated activation of ATF2 contributes to dopaminergic neurodegeneration in the MPTP mouse model of Parkinson’s disease. Exp. Neurol. 277, 296–304. 10.1016/j.expneurol.2015.10.010 26515688

[B37] Hurtado D. E. Molina-Porcel L. Carroll J. C. Macdonald C. Aboagye A. K. Trojanowski J. Q.. (2012). Selectively silencing GSK-3 isoforms reduces plaques and tangles in mouse models of Alzheimer’s disease. J. Neurosci. 32, 7392–7402. 10.1523/jneurosci.0889-12.2012 22623685 PMC3368584

[B39] Kaidanovich-Beilin O. Lipina T. V. Takao K. van Eede M. Hattori S. Laliberte C.. (2009). Abnormalities in brain structure and behavior in GSK-3α mutant mice. Mol. Brain 2:35. 10.1186/1756-6606-2-35 19925672 PMC2785804

[B38] Kaidanovich-Beilin O. Woodgett J. R. (2011). GSK-3: functional insights from cell biology and animal models. Front. Mol. Neurosci. 4:40. 10.3389/fnmol.2011.00040 22110425 PMC3217193

[B40] Kalia L. V. Lang A. E. (2015). Parkinson’s disease. Lancet 386, 896–912. 10.1016/S0140-6736(14)61393-3 25904081

[B41] Kim D. W. Lee J. H. Park S. K. Yang W. M. Jeon G. S. Lee Y. H.. (2007). Astrocytic expressions of phosphorylated Akt, GSK3β and CREB following an excitotoxic lesion in the mouse hippocampus. Neurochem. Res. 32, 1460–1468. 10.1007/s11064-007-9332-y 17417726

[B42] Kim W. Won S. Y. Yoon B. J. (2019). CRMP2 mediates GSK3β actions in the striatum on regulating neuronal structure and mania-like behavior. J. Affect. Disord. 245, 1079–1088. 10.1016/j.jad.2018.10.371 30699850

[B43] Leng Y. Liang M. H. Ren M. Marinova Z. Leeds P. Chuang D. M. (2008). Synergistic neuroprotective effects of lithium and valproic acid or other histone deacetylase inhibitors in neurons: roles of glycogen synthase kinase-3 inhibition. Proc. Natl. Acad. Sci. U S A 28, 2576–2588. 10.1523/jneurosci.5467-07.2008 18322101 PMC5241911

[B44] Li N. Qiao M. Zhang P. Li X. Li L. Yu Z. (2016). The effects of early life lead exposure on the expression of glycogen synthase kinase-3β and insulin-like growth factor 1 receptor in the hippocampus of mouse pups. Biol. Trace Elem. Res. 169, 114–120. 10.1007/s12011-015-0382-8 26085056

[B45] Li X. Rosborough K. M. Friedman A. B. Zhu W. Roth K. A. (2007). Regulation of mouse brain glycogen synthase kinase-3 by atypical antipsychotics. Int. J. Neuropsychopharmacol. 10, 7–19. 10.1017/s1461145706006547 16672106

[B46] Li Y. Xu M. Ding X. Yan C. Song Z. Chen L.. (2016). Protein kinase C controls lysosome biogenesis independently of mTORC1. Nat. Cell Biol. 18, 1065–1077. 10.1038/ncb3407 27617930

[B47] Liang M. H. Chuang D. M. (2007). Regulation and function of glycogen synthase kinase-3 isoforms in neuronal survival. J. Biol. Chem. 282, 3904–3917. 10.1074/jbc.m605178200 17148450

[B48] Lin C. H. Lin H. I. Chen M. L. Lai T. T. Cao L. P. Farrer M. J.. (2016). Lovastatin protects neurite degeneration in LRRK2–G2019S parkinsonism through activating the Akt/Nrf pathway and inhibiting GSK3β activity. Hum. Mol. Genet. 25, 1965–1978. 10.1093/hmg/ddw068 26931464

[B49] Lovestone S. Boada M. Dubois B. Hull M. Rinne J. O. Huppertz H. J.. (2015). A phase II trial of tideglusib in Alzheimer’s disease. J. Alzheimers Dis. 45, 75–88. 10.3233/JAD-141959 25537011

[B50] MacAulay K. Doble B. W. Patel S. Hansotia T. Sinclair E. M. Drucker D. J.. (2007). Glycogen synthase kinase 3α-specific regulation of murine hepatic glycogen metabolism. Cell Metab. 6, 329–337. 10.1016/j.cmet.2007.08.013 17908561

[B51] Mann A. Chesselet M.-F. (2015). “Chapet 8-Techniques for motor assessment in rodents,” in Movement DisordersGenetics and Models, 2nd Edn., ed. LeDoux M. (San Diego, CA: Academic Press), 139–157. 10.1016/B978-0-12-405195-9.00008-1

[B52] Marchand B. Arsenault D. Raymond-Fleury A. Boisvert F. M. Boucher M. J. (2015). Glycogen synthase kinase-3 (GSK3) inhibition induces prosurvival autophagic signals in human pancreatic cancer cells. J. Biol. Chem. 290, 5592–5605. 10.1074/jbc.m114.616714 25561726 PMC4342473

[B53] Martin M. Rehani K. Jope R. S. Michalek S. M. (2005). Toll-like receptor-mediated cytokine production is differentially regulated by glycogen synthase kinase 3. Nat. Immunol. 6, 777–784. 10.1038/ni1221 16007092 PMC1933525

[B54] Mathuram T. L. Reece L. M. Cherian K. M. (2018). GSK-3 inhibitors: a double-edged sword?—An update on Tideglusib. Drug Res. 68, 436–443. 10.1055/s-0044-100186 29388174

[B55] Matsunaga S. Fujishiro H. Takechi H. (2019). Efficacy and safety of glycogen synthase kinase-3 inhibitors for Alzheimer’s disease: a systematic review and meta-analysis. J. Alzheimers Dis. 69, 1031–1039. 10.3233/jad-190256 31156177

[B56] Meredith G. E. Totterdell S. Petroske E. Santa Cruz K. Callison R. C. Jr. Lau Y. S. (2002). Lysosomal malfunction accompanies α-synuclein aggregation in a progressive mouse model of Parkinson’s disease. Brain Res. 956, 156–165. 10.1016/s0006-8993(02)03514-x 12426058

[B57] Morales-Garcia J. A. Susin C. Alonso-Gil S. Perez D. I. Palomo V. Perez C.. (2013). Glycogen synthase kinase-3 inhibitors as potent therapeutic agents for the treatment of Parkinson disease. ACS Chem. Neurosci. 4, 350–360. 10.1021/cn300182g 23421686 PMC3582296

[B58] Morgan-Smith M. Wu Y. Zhu X. Pringle J. Snider W. D. (2014). GSK-3 signaling in developing cortical neurons is essential for radial migration and dendritic orientation. Elife 3:e02663. 10.7554/elife.02663 25073924 PMC4109311

[B59] Nagao M. Hayashi H. (2009). Glycogen synthase kinase-3β is associated with Parkinson’s disease. Neurosci. Lett. 449, 103–107. 10.1016/j.neulet.2008.10.104 19007860

[B60] Noori M. S. Bhatt P. M. Courreges M. C. Ghazanfari D. Cuckler C. Orac C. M.. (2019). Identification of a novel selective and potent inhibitor of glycogen synthase kinase-3. Am. J. Physiol. Cell Physiol. 317, C1289–C1303. 10.1152/ajpcell.00061.2019 31553649 PMC6962522

[B61] Nuutinen U. Ropponen A. Suoranta S. Eeva J. Eray M. Pellinen R.. (2009). Dexamethasone-induced apoptosis and up-regulation of Bim is dependent on glycogen synthase kinase-3. Leuk. Res. 33, 1714–1717. 10.1016/j.leukres.2009.06.004 19559478

[B62] Papazoglou I. K. Jean A. Gertler A. Taouis M. Vacher C. M. (2015). Hippocampal GSK3β as a molecular link between obesity and depression. Mol. Neurobiol. 52, 363–374. 10.1007/s12035-014-8863-x 25169083

[B63] Patel S. Doble B. W. MacAulay K. Sinclair E. M. Drucker D. J. Woodgett J. R. (2008). Tissue-specific role of glycogen synthase kinase 3β in glucose homeostasis and insulin action. Mol. Cell. Biol. 28, 6314–6328. 10.1128/mcb.00763-08 18694957 PMC2577415

[B64] Paxinos G. Franklin K. B. (2001). The Mouse Brain in Stereotaxic Coordinates. San Diego, CA: Academic Press.

[B65] Ren Y. Chen J. Wu X. Gui C. Mao K. Zou F.. (2018). Role of c-Abl-GSK3β signaling in MPP^+^-induced autophagy-lysosomal dysfunction. Toxicol. Sci. 165, 232–243. 10.1093/toxsci/kfy155 30165626

[B66] Roh M. S. Eom T. Y. Zmijewska A. A. De Sarno P. Roth K. A. Jope R. S. (2005). Hypoxia activates glycogen synthase kinase-3 in mouse brain *in vivo*: protection by mood stabilizers and imipramine. Biol. Psychiatry 57, 278–286. 10.1016/j.biopsych.2004.10.039 15691529

[B67] Rubio-Patiño C. Palmeri C. M. Perez-Perarnau A. Cosialls A. M. Moncunill-Massaguer C. Gonzalez-Girones D. M.. (2012). Glycogen synthase kinase-3β is involved in ligand-dependent activation of transcription and cellular localization of the glucocorticoid receptor. Mol. Endocrinol. 26, 1508–1520. 10.1210/me.2011-1366 22771494 PMC5416974

[B68] Russell J. C. Kishimoto K. O’Driscoll C. Hossain M. A. (2011). Neuronal pentraxin 1 induction in hypoxic-ischemic neuronal death is regulated *via* a glycogen synthase kinase-3α/β dependent mechanism. Cell. Signal. 23, 673–682. 10.1016/j.cellsig.2010.11.021 21130869 PMC3056287

[B69] Serenó L. Coma M. Rodríguez M. Sánchez-Ferrer P. Sánchez M. B. Gich I.. (2009). A novel GSK-3β inhibitor reduces Alzheimer’s pathology and rescues neuronal loss *in vivo*. Neurobiol. Dis. 35, 359–367. 10.1016/j.nbd.2009.05.025 19523516

[B70] Song B. Lai B. Zheng Z. Zhang Y. Luo J. Wang C.. (2010). Inhibitory phosphorylation of GSK-3 by CaMKII couples depolarization to neuronal survival. J. Biol. Chem. 285, 41122–41134. 10.1074/jbc.m110.130351 20841359 PMC3003410

[B71] Sutherland C. Cohen P. (1994). The α-isoform of glycogen synthase kinase-3 from rabbit skeletal muscle is inactivated by p70 S6 kinase or MAP kinase-activated protein kinase-1 *in vitro*. FEBS Lett. 338, 37–42. 10.1016/0014-5793(94)80112-6 8307153

[B72] Sutherland C. Leighton I. A. Cohen P. (1993). Inactivation of glycogen synthase kinase-3 β by phosphorylation: new kinase connections in insulin and growth-factor signalling. Biochem. J. 296, 15–19. 10.1042/bj2960015 8250835 PMC1137648

[B73] Tolosa E. Litvan I. Hoglinger G. U. Burn D. Lees A. Andres M. V.. (2014). A phase 2 trial of the GSK-3 inhibitor tideglusib in progressive supranuclear palsy. Mov. Disord. 29, 470–478. 10.1002/mds.25824 24532007

[B74] Vila M. Bové J. Dehay B. Rodríguez-Muela N. Boya P. (2011). Lysosomal membrane permeabilization in Parkinson disease. Autophagy 7, 98–100. 10.4161/auto.7.1.13933 21045565

[B77] Wang Y. H. Liou K. T. Tsai K. C. Liu H. K. Yang L. M. Chern C. M.. (2018). GSK-3 inhibition through GLP-1R allosteric activation mediates the neurogenesis promoting effect of P7C3 after cerebral ischemic/reperfusional injury in mice. Toxicol. Appl. Pharmacol. 357, 88–105. 10.1016/j.taap.2018.08.023 30189238

[B76] Wang Y. Liu W. He X. Zhou F. (2013). Parkinson’s disease-associated DJ-1 mutations increase abnormal phosphorylation of tau protein through Akt/GSK-3β pathways. J. Mol. Neurosci. 51, 911–918. 10.1007/s12031-013-0099-0 23979838

[B75] Wang W. Yang Y. Ying C. Li W. Ruan H. Zhu X.. (2007). Inhibition of glycogen synthase kinase-3β protects dopaminergic neurons from MPTP toxicity. Neuropharmacology 52, 1678–1684. 10.1016/j.neuropharm.2007.03.017 17517424

[B78] Wills J. Jones J. Haggerty T. Duka V. Joyce J. N. Sidhu A. (2010). Elevated tauopathy and α-synuclein pathology in postmortem Parkinson’s disease brains with and without dementia. Exp. Neurol. 225, 210–218. 10.1016/j.expneurol.2010.06.017 20599975 PMC2922478

[B79] Woodgett J. R. (1990). Molecular cloning and expression of glycogen synthase kinase-3/factor A. EMBO J. 9, 2431–2438. 10.1002/j.1460-2075.1990.tb07419.x 2164470 PMC552268

[B80] Yang K. Chen Z. Gao J. Shi W. Li L. Jiang S.. (2017). The key roles of GSK-3β in regulating mitochondrial activity. Cell Physiol. Biochem. 44, 1445–1459. 10.1159/000485580 29190615

[B81] Yu Q. Huang Q. Du X. Xu S. Li M. Ma S. (2018). Early activation of Egr-1 promotes neuroinflammation and dopaminergic neurodegeneration in an experimental model of Parkinson’s disease. Exp. Neurol. 302, 145–154. 10.1016/j.expneurol.2018.01.009 29337144

[B82] Yuskaitis C. J. Jope R. S. (2009). Glycogen synthase kinase-3 regulates microglial migration, inflammation and inflammation-induced neurotoxicity. Cell. Signal. 21, 264–273. 10.1016/j.cellsig.2008.10.014 19007880 PMC2630396

